# Case Report: Celiac plexus block improves gastrointestinal Long COVID symptoms

**DOI:** 10.3389/fnins.2025.1589809

**Published:** 2025-11-21

**Authors:** Luke D. Liu, Deborah L. Duricka

**Affiliations:** 1 Neuroversion Inc., Anchorage, AK, United States; 2 WWAMI School of Medical Education, Anchorage, AK, United States

**Keywords:** Long COVID, irritable bowel syndrome (IBS), gastrointestinal (GI) dysfunction, visceral hyperalgesia, diarrhea, constipation, stellate ganglion block (SGB), celiac plexus block (CPB)

## Abstract

Lingering symptoms following SARS-CoV-2 infection, recognized as the clinical entity “Long COVID,” are common. Gastrointestinal dysfunction during and after COVID have received little attention to date and remain largely unaddressed. We have previously shown that numerous symptoms of Long COVID excluding gastrointestinal symptoms improve or resolve following stellate ganglion blocks (SGB). Here, we are first to report successful treatment of persistent post-COVID epigastric pain and diarrhea in three patients using celiac plexus block, a procedure commonly used for visceral abdominal pain and implicating the autonomic nervous system in Long COVID-associated GI symptoms.

## 1 Introduction

Following the SARS-CoV-2 pandemic, millions of individuals suffer from Long COVID—a constellation of symptoms including gastrointestinal (GI) dysfunction with unknown pathophysiology (Perumal et al., 2023). A recent case-controlled study showed a twofold increased risk of loose stools 5 months after recovery from COVID-19, especially when diarrhea was experienced during the acute infection (Noviello et al., 2022). A population-based cohort study using the UK Biobank database (*n* = 112,311 and *n* = 370,979) with median follow-up of 0.7 years found hazard ratios of 1.38 for GI dysfunction, and 1.36 for pancreatic disease which increased to 2.57 with multiple COVID infections (Ma et al., 2024) A cohort study utilizing the U.S. Department of Veteran Affairs’ database (*n* = 154,068 and *n* = 5,638,795) found a hazard ratio of 1.54 for irritable bowel syndrome (IBS) 1 year after COVID infection (Xu et al., 2023). The pooled prevalence for IBS following COVID-19 is 15% across various countries according to a recent meta-analysis (Wang et al., 2023). Infection and gut inflammation can result in the clinical syndrome termed Post-Infectious IBS (PI-IBS), which is characterized by persistent neuroplasticity and gut dysfunction (Brierley and Linden, 2014). While the enteric nervous system (ENS) can function autonomously, normal gut function relies not only on coordination of intrinsic ENS neurons but also on communication of extrinsic neurons with central nervous system (CNS) neurons, which together form the gut-brain axis. Ordinarily, the gut-brain axis integrates ascending information from the gut and enables higher-order brain functions to influence peripheral processes, including intestinal activity and immune activation (Carabotti et al., 2015). Deeply located in the retroperitoneum, the celiac ganglia, the superior mesenteric ganglia, and the aorticorenal ganglia comprise the celiac plexus (CP), which is an essential hub in the gut-brain axis. Organs receiving autonomic (sympathetic efferent) innervation via the CP and whose visceral afferent fibers synapse at the CP before ascending to higher-order centers in the CNS include the stomach, liver, gallbladder, pancreas, kidneys, spleen, small bowel, and the first two-thirds of the large bowel. In animal models, the coordinated firing of myenteric neurons is transmitted by interneurons in the gut wall to prevertebral ganglia, where sympathetic reflex activation occurs without preganglionic input (Hibberd et al., 2020). In the context of dysfunction following remodeling during inflammation, this feedback loop can potentially amplify pain signals and maintain abnormal gut motility induced during acute infection.

The first known mention of celiac plexus block (CPB) in the literature describes its use in surgical anesthesia by Kappis (1914). Since then, its applications have expanded to include CP block and CP neurolysis as effective treatments for chronic pancreatitis and pancreatic cancer pain (Cornman-Homonoff et al., 2017). It can be guided by anatomic landmarks, fluoroscopy, ultrasound, computed tomography, or magnetic resonance imaging. A variety of approaches (retrocrural, transcrural, transaortic, preaortic, transdiscal, and endoscopic) and widely variable volumes of anesthetic solution (15–50 mL on each side) have been used to achieve effective blockade of the entire celiac plexus (Cornman-Homonoff et al., 2017; Kambadakone et al., 2011; Pribonic et al., 2023; Urits et al., 2020; Vig et al., 2021). Standardizing the procedure is inadvisable due to significant anatomical variations (Pereira et al., 2014). Even with imaging guidance, its potential risks include pneumothorax and traumatic penetrating injuries of nearby visceral organs (Yang et al., 2011). Nevertheless, it is considered generally safe, with serious complications reported in 0.14% in a large retrospective study in 1993 (Davies, 1993). Common side effects include transient orthostatic hypotension (10%–52% of patients) and diarrhea that typically resolves within 48 h (44%–60% of patients) while durable pain relief has been reported in 70%–90% of cancer patients with neurolytic agents (Gupta et al., 2021).

Previously, we reported sequential bilateral stellate ganglion blocks (SGB) as a novel effective treatment for Long COVID symptoms including orthostatic intolerance, fatigue, anxiety, and depression (Duricka and Liu, 2024, 2025; Liu and Duricka, 2022). Since then, we observed that some patients experience persistent GI complaints (including epigastric pain, diarrhea, and constipation) unresponsive to SGB. To our knowledge, we report for the first time the sustained improvement of Long COVID-associated gastrointestinal symptoms in three patients using celiac plexus block (CPB), a technique historically used for intractable visceral abdominal pain (Cornman-Homonoff et al., 2017) as an adjunct to SGB for Long COVID patients with refractory GI dysfunction.

## 2 Case description

All three patients were females between the ages of 33 and 39 who had recovered from mild acute COVID infection 6 to 14 months prior to presentation (Table 1). Each suffered from continuing diarrhea since recovering from COVID, despite normal GI workups and no prior history of abdominal issues or chronic illness. They also reported post-COVID symptoms of orthostatic intolerance, fatigue, and increased anxiety.

**Table 1:** Demographic data, vitals, Long COVID details, and current medications for each patient before treatment.

ID	Age	BP, HR, Temp	BMI	O_2_ Sat	# of COVID infections	Duration of symptoms	Current medications
1	35	118/77, 67, 98.4	24.5	98	1	14 months	NAC, Armour Thyroid, Naltrexone
2	32	166/110, 66, 98.7	27.6	95	1	6 months	Fenofibrate, dicyclomine, sucralfate, promethazine, nortriptyline, alosetron, aripiprazole, rizatriptan, Zofran, Zoloft, Trileptal
3	39	117/75, 79, 97.3	31.3	95	4	35 months	Lorazepam, rizatriptan, Restasis, Ajovy

ID, Patient #1–3; BP, blood pressure (mmHg); HR, heart rate (beats per minute); Temp, temperature (°F); BMI, body mass index; O_2_ Sat, oxygen saturation (%) on room air; Duration of symptoms, time from first COVID infection to presentation at clinic.

## 3 Timeline

### 3.1 Diagnostic assessment, therapeutic interventions, outcomes

Upon examination, vital signs were within normal range for subjects 1 and 3, while subject 2 displayed hypertension (Table 1). General physical exams were unremarkable, except for epigastric tenderness and bilateral cold, clammy hands. Subjects used the 0–10 numeric rating scale (NRS) to establish the degree of baseline bowel changes compared to their pre-COVID habits (diarrhea in each case), dizziness upon standing, and rapid heartbeat. Fatigue was assessed using the PROMIS SF-7a (Patient Reported Outcomes Short Form version 1.0 Fatigue 7a), a 7-item questionnaire validated for measuring fatigue associated with inflammatory bowel disease (IBD) (Feagan et al., 2023), which shares similarities with and can follow post-viral GI dysfunction (Brierley and Linden, 2014). Depression was assessed using the PHQ-9 (Patient Health Questionnaire-9), a 9-item questionnaire designed to screen for the presence and severity of depressive symptoms that has been validated in patients with IBD (Litster et al., 2018) and in patients with irritable bowel syndrome (IBS) (Snijkers et al., 2021). Anxiety was measured using the GAD-7 (Generalized Anxiety Disorder-7) assessment tool, a 7-item questionnaire used to screen for generalized anxiety disorder and symptom severity that has been validated in patients with IBS (Snijkers et al., 2021). Scores for PROMIS SF-7a, PHQ-9, and GAD-7 were normalized to the 0–10 scale used to measure bowel changes, dizziness upon standing, and rapid heartbeat. Assessments were diagnostic for significant bowel changes, fatigue, anxiety, and depression.

**Figure 1:**
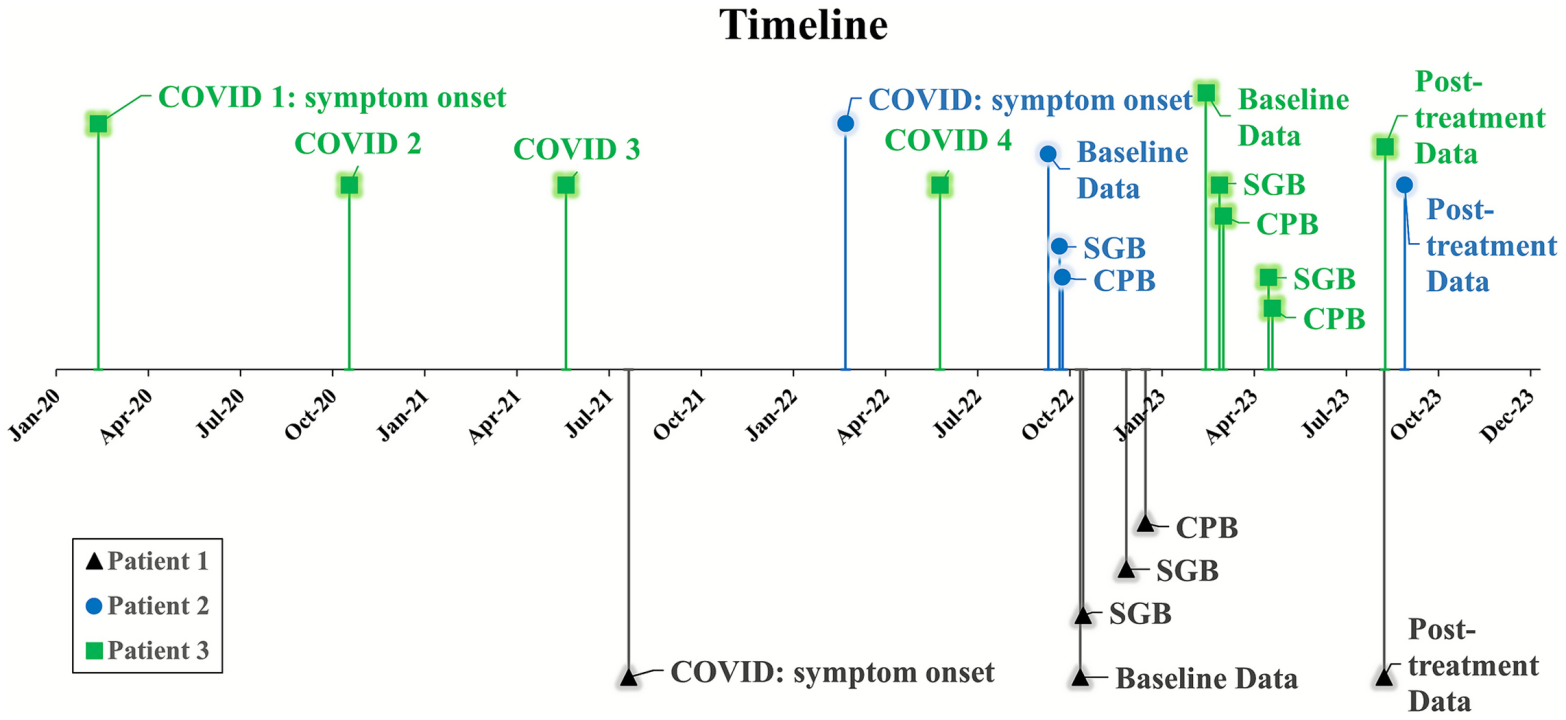
Timeline of events. SGB, sequential bilateral stellate ganglion blocks performed within 24 h of each other. CPB, celiac plexus block. COVID, confirmed SARS-CoV-2 infection. Patient 3 was infected four times with SARS-CoV-2, indicated by numbers 1–4.

**Figure 2:**
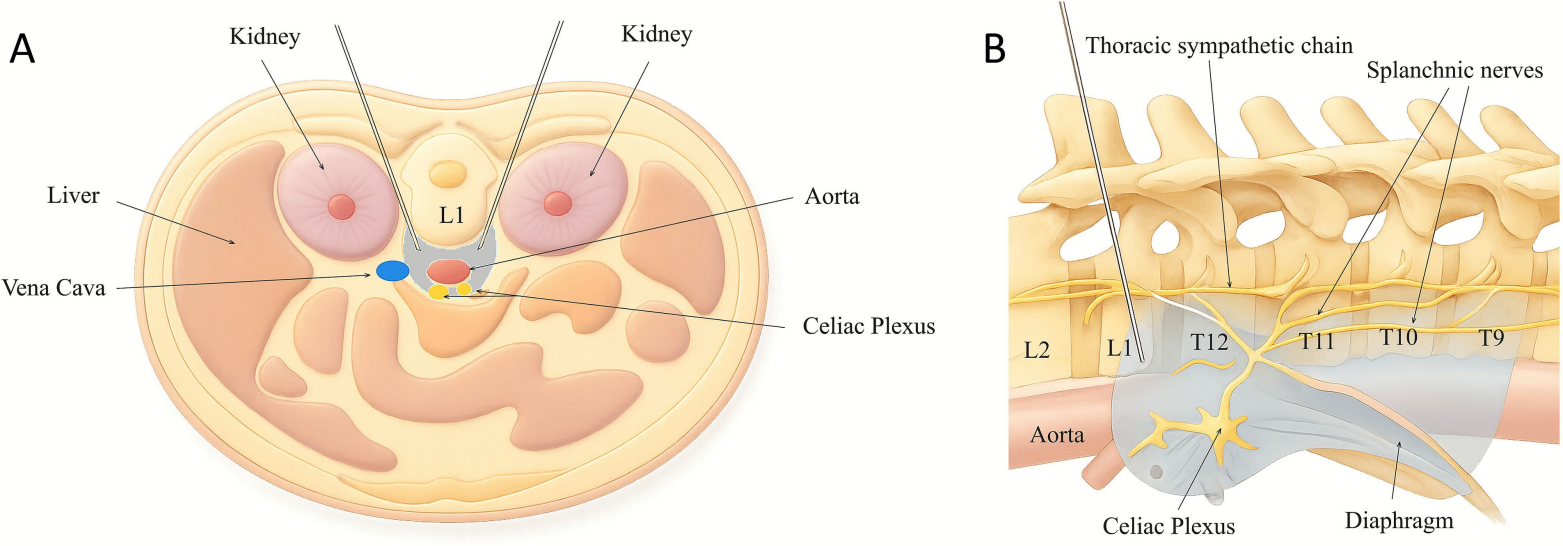
Diagrams illustrating the posterior retrocrural approach for celiac plexus block. **(A)** Axial view with both needles in place. **(B)** Lateral view (prone) with one needle in place. Gray area indicates spread of injectate. T, thoracic vertebra; L, lumbar vertebra.

**Figure 3:**
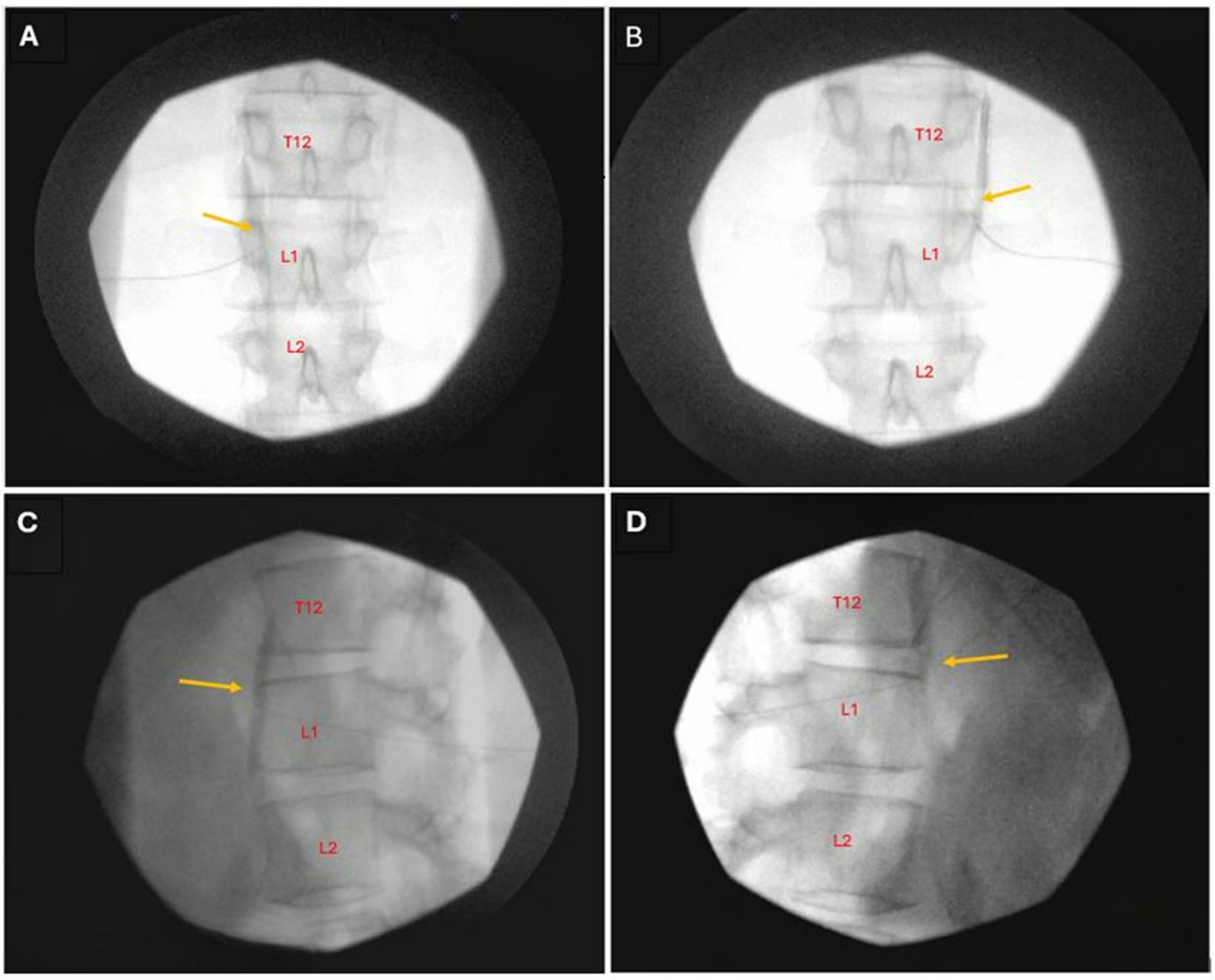
Radiographs demonstrating the posterior retrocrural approach for celiac plexus block. **(A,C)** Left-sided approach. **(B,D)** Right-sided approach. **(A,B)** Anteroposterior view. **(C,D)** Lateral view. T, thoracic vertebra; L, lumbar vertebra. Yellow arrows indicate contrast spread at injection site.

Each patient received sequential bilateral SGBs, as previously described (Duricka and Liu, 2025). Briefly, under ultrasound guidance, a 27-gauge 2-inch hypodermic needle was used to administer 10 mL bupivacaine 0.5% around the stellate ganglion site at C7 level. Horner’s syndrome was observed and documented. Subsequently, 1 to 30 days after SGBs, the patients received CPBs (see Figure 1 for timeline). To prevent expected and clinically significant hypotension upon successful block of the celiac plexus, 1–2 L normal saline IV bolus was provided preemptively. Each CPB was performed using fluoroscopy guided posterior bilateral retrocrural approach (Pribonic et al., 2023) (Figures 2, 3), in which two 25-gauge 7-inch spinal needles were advanced bilaterally onto the anterolateral aspect of superior L1 vertebral body under fluoroscopy guidance. After negative aspiration, 0.5–1.0 mL iodinated contrast agent was injected to confirm the expected cephalocaudal spread along the anterolateral portion of the thoracolumbar junction. Then, 20 mL of Bupivacaine 0.25% was injected on each side to achieve effective CPB.

**Figure 4:**
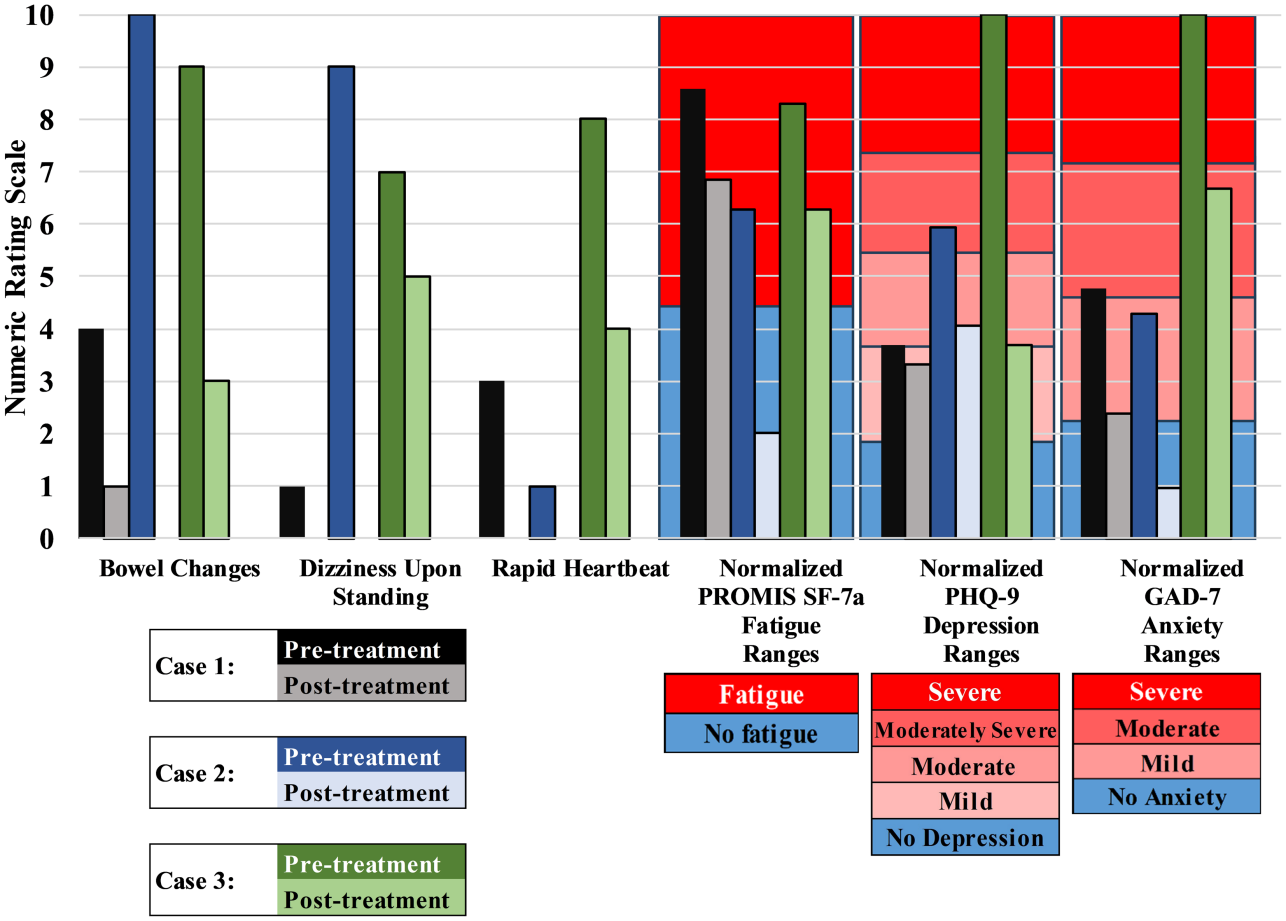
Effect of treatment on Long COVID symptoms of patients. Treatment reduced symptoms of diarrhea (“bowel changes”), dizziness upon standing, rapid heartbeat, fatigue, depression, and anxiety for all three patients. See Figure 1 for timeline. Higher scores indicate more severe symptoms. Patients were asked to estimate their bowel changes (compared to pre-COVID baseline), dizziness upon standing, and rapid heartbeat on a scale of 1–10. PROMIS SF-7a, Patient-Reported Outcomes Measurement Information System Short Form version 1.0 – Fatigue 7a (a validated tool for measuring fatigue); PHQ-9, Patient Health Questionnaire-9 (a validated tool for measuring depression); GAD-7, Generalized Anxiety Disorder-7 (a validated tool for measuring anxiety). PROMIS SF-7a, PHQ-9, GAD-7, and their respective cutoff scores were normalized to allow for comparison with bowel changes, dizziness upon standing, and rapid heartbeat.

Patients 1–3 reported 100%, 80%, and 70% relief of abdominal pain, respectively, at two-week follow-up. Assessments for bowel changes, dizziness upon standing, rapid heartbeat, fatigue, depression, and anxiety were repeated 6 to 12 months after treatment (Figures 1, 4). At the time of writing (15 to 21 months after treatment), Patients 1 & 2 report durable relief of GI symptoms, while Patient 3 requires repeat procedures every 3 months to maintain benefit.

## 4 Discussion

Reports of GI symptoms during acute SARS-CoV-2 infection range from 10% to 40% (Delgado-Gonzalez et al., 2021). In animal models of infection-induced intestinal inflammation, visceral hyperalgesia is exacerbated by stress and anxiety, and is maintained by persistent neuroplastic changes even after the resolution of infection (Brierley and Linden, 2014). Additionally, studies have shown that visceral hypersensitivity induces anxiety behavior, creating a positive feedback loop. Notably, the underlying neuronal pathway requires less stimulus in females (Bayrer et al., 2023) which may contribute to the gender disparity in Long COVID prevalence.

All three patients reported GI symptoms including diarrhea during the acute phase of COVID-19 that persisted throughout Long COVID despite normal GI workups. After CPB, patients reported durable 70%–100% relief of abdominal pain, along with improvement in bowel function and a reduction of diarrhea for up to 21 months (Figures 1, 4). Notably, patient 3 has required repeat treatment at approximately 3-month intervals to maintain symptom relief. Uniquely in this case series, this patient contracted COVID three additional times after the initial SARS-CoV-2 infection and onset of Long COVID (Figure 1). It is well established that each SARS-CoV-2 infection increases the cumulative risk of developing Long COVID (Bowe et al., 2022; Romero-Ibarguengoitia et al., 2024), and preliminary research (preprint) suggests that Long COVID severity and persistence worsen with multiple infections (Soares et al., 2024), which may explain the recalcitrance of this patient’s symptoms.

While sympathetic hyperactivity is generally associated with constipation, a population-based study found an association between indices of sympathetic activation, autonomic dysfunction, and functional diarrhea (Hamrefors et al., 2019). CPB shows promise as a treatment for Long COVID-related functional GI symptoms, potentially by normalizing the autonomic tone of the gut-brain axis and modulating neuroimmunological processes through reduced sympathetic hyperresponsiveness. Additional or alternative mechanisms contributing to GI symptom relief may include improved tissue perfusion and lymphatic flow−both known effects of sympathectomy in pathological conditions such as breast cancer-related lymphedema and complex regional pain syndrome (Choi et al., 2015; Howarth et al., 1999; Park et al., 2015). Indeed, lymphatic and vascular dysfunction persist after acute small intestine inflammation and are hallmarks of IBD, particularly Crohn’s disease (Alexander et al., 2010; Randolph et al., 2016; Rehal et al., 2018). Although CPB is offered by some providers for the treatment of IBS and IBD, its full therapeutic potential remains largely unexplored. While its role in providing visceral analgesia is recognized, it is unclear whether CPB confers broader benefits, such as modulating autonomic dysfunction, reducing neuroinflammatory processes, or improving gastrointestinal motility and barrier function. Further research is needed to determine whether CPB can influence the underlying pathophysiology of these conditions beyond symptom relief, potentially offering a novel approach to managing functional and inflammatory GI disorders. We noted improvements in rapid heartbeat, dizziness upon standing, anxiety, depression, and fatigue in all three patients, consistent with our previous studies utilizing SGB for Long COVID (Duricka and Liu, 2024, 2025; Liu and Duricka, 2022). However, it remains unclear whether these positive clinical responses observed in this case series were due to SGB, CPB, or a combination of both, and whether their effects are independent, additive, or synergistic. It is plausible that alleviation of GI symptoms following CPB could enhance the benefits of SGB on non-GI symptoms.

Functional GI disorders constitute a substantial unmet medical need, diminishing quality of life and daily activities and, in some cases, causing profound disability. Consistent with the nature of a case series, there are significant limitations to extrapolation of our data, including small sample size and subjective assessments. Nonetheless, by reporting our treatment success, we aim to expand the therapeutic armamentarium and stimulate research for Long COVID and other nonstructural GI dysfunctions. Confirmatory studies and mechanistic investigations into how regional sympathectomy improves Long COVID symptoms are warranted.

## 5 Patient perspective

Patient 1: My abdominal symptoms have greatly improved since the celiac plexus block. I have not experienced an episode of unbearable abdominal pain since the treatment.

Patient 2: I no longer have diarrhea! My bowel movements are more regular and less frequent.

Patient 3: This treatment allowed me to be a mom, businesswoman, and wife again.

## Data Availability

The raw data supporting the conclusions of this article will be made available by the authors, without undue reservation.
